# Treatment of upper limb arterial occlusion caused by a cervical rib

**DOI:** 10.1590/1677-5449.200193

**Published:** 2021-06-16

**Authors:** Vanessa Aline Miranda Vieira Milagres, Roberto Lucas de Sena Avellar, Ana Paula Pires Silva, Pedro José Pires, Daniel Mendes Pinto

**Affiliations:** 1 Hospital Felício Rocho, Belo Horizonte, MG, Brasil.

**Keywords:** cervical rib, thoracic outlet syndrome, subclavian artery, subclavian vein, brachial plexus

## Abstract

The cervical rib syndrome occurs when the interscalene triangle is occupied by a cervical rib, displacing the brachial plexus and the subclavian artery forward, which can cause pain and muscle spasms. The objective of this study is to discuss diagnosis of the cervical rib syndrome and treatment possibilities. This therapeutic challenge describes clinical and surgical management of a 37-year-old female patient with upper limb arterial occlusion caused by a cervical rib.

## INTRODUCTION

A cervical rib is an anatomic variant that is present in 1% of the population.^1^ It originates from the transverse process of the seventh cervical vertebra.^1^ The majority of patients are asymptomatic and just 10% manifest symptoms.^1^ When present, symptoms are caused by compression of neurovascular structures in the thoracic outlet region, constituting the cervical rib syndrome, which is one type of thoracic outlet syndrome (TOS). A cervical rib is present in almost 30% of cases of TOS.^2^

Surgical treatment of the cervical rib syndrome can be performed via conventional access routes, such as the supraclavicular, posterior, transaxillary, or combined approaches.^3^^,^^4^ More recently described approaches include video-assisted surgery and transthoracic with robotic assistance.^5^^,^^6^

The objective of this study is to discuss diagnosis of cervical rib syndrome and the treatment possibilities. We present the case of a 37-year-old female patient with an upper limb arterial obstruction caused by a cervical rib and discuss its clinical and surgical management. The protocol was approved by the Ethics Committee at our institution (CAAE 35649620.2.0000.5125, approval ruling no. 4.303.586).

## PART I – CLINICAL SITUATION

A 37-year-old, previously healthy, female patient was admitted to an Urgent Care Center in September 2019 with pain, pallor, and paresthesia in the right upper limb. She reported onset of symptoms 4 months previously, with deterioration over the last 2 weeks. Physical examination of the right upper limb found distal pallor, a palpable pulse in the right supraclavicular region, weak brachial pulse, and absent radial and ulnar pulses. There was a palpable cervical rib on the right. Arterial duplex ultrasound of the right upper limb revealed thrombi with a chronic appearance in the radial and ulnar arteries, with occlusion. Radiography of the cervical spine and thorax showed an articulated cervical rib on the right. This situation raised a number of treatment options:

1- Systemic anticoagulation with heparin;
2- Catheter-guided thrombolysis;
3- Surgery to resect the cervical rib.

## PART II – WHAT WAS DONE

**Figure 1:**
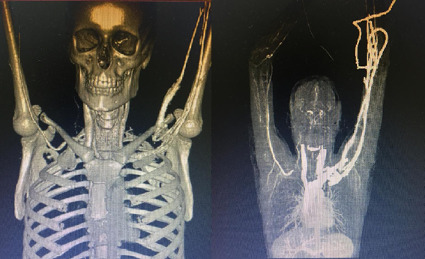
Angiotomography showing the cervical rib on the right and compression of the subclavian artery.

The patient was admitted and given pain control and anticoagulation with enoxaparin at 1 mg/kg every 12 h. Angiotomography of the thoracic aorta and the right upper limb identified an accessory cervical rib (C7) on the right, joining to the first ipsilateral rib anteriorly. The subclavian artery was patent, but subjected to considerable compression between the accessory rib described above and the ipsilateral clavicle, increasing notably during abduction of the right upper limb (Figure 1). Other patent segments of the right subclavian artery did not exhibit evidence of compression. Having established a diagnosis of TOS, the patient was discharged on warfarin and acetylsalicylic acid, with an international normalized ratio result of 2.39, and was instructed to attend outpatients follow-up and schedule cervical rib resection surgery.

**Figure 2:**
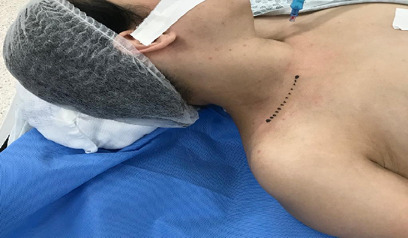
“Necklace” incision, 2 cm superior of the clavicle.

**Figure 3:**
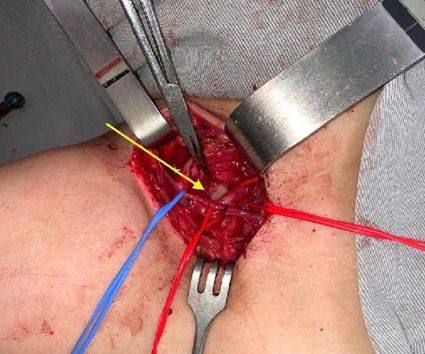
The yellow arrow indicates the cervical rib. Repair of the subclavian artery with a red vessel loop.

After 1 month on therapeutic anticoagulation, in October 2019, the patient was admitted for elective surgical treatment of TOS. Access was obtained via a supraclavicular incision, as illustrated in Figure 2, with identification of the cervical rib, the subclavian artery, and the brachial plexus (Figure 3).

**Figure 4:**
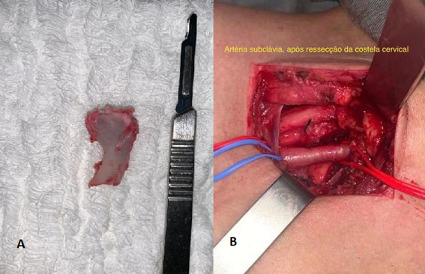
(A) Cervical rib; (B) Subclavian artery after resection of the cervical rib.

The patient underwent resection of the right cervical rib (Figure 4), with dissection and release of adhesions to the right subclavian artery and exploration of the brachial plexus. The surgical operation was well-tolerated and was conducted with no intraoperative complications. Perioperative chest X-ray did not show pneumothorax. During the postoperative period, the patient recovered well, with improvement in pain, conservation of upper limb sensitivity and motricity, and full and symmetrical radial pulses. She was discharged on the first postoperative day on 100 mg of acetylsalicylic acid per day. At 6 months’ follow-up, she was asymptomatic and had good perfusion of the right upper limb.

## DISCUSSION

This study draws attention to arterial occlusion in young patients, which can be caused by mechanical compression. About 50% of patients with cervical rib syndrome present with arterial compression.^2^ Treatment via a cervical approach is appropriate in this situation, because it enables safe access to the brachial plexus, the subclavian artery, and the cervical rib.

Thoracic outlet syndrome encompasses symptoms caused by compression of neurovascular structures in the region of the thoracic outlet.^5^ Cervical rib syndrome occurs when the interscalene triangle is occupied by a cervical rib, displacing the brachial plexus and subclavian artery forward, causing pain and muscle spasms.^1^

Treatment of the majority of patients with TOS is clinical, involving analgesics, anti-inflammatories, benzodiazepines, and postural changes.^1^ In the case presented here, clinical treatment was initiated with analgesics and therapeutic anticoagulation because the patient had presented with arterial occlusion. There are formal indications for surgery in 15% of cases and the majority of operations to treat TOS are conducted in patients with neurogenic compression.^1^ Presence of cervical rib, symptomatic bone abnormalities, and vascular complications such as aneurysms and thromboses, are indications for mandatory surgery.^1^^,^^2^ According to Daniels et al.,^3^ when thrombus is present, catheter-guided thrombolysis is the initial treatment of choice. In this case, we decided to initiate treatment with anticoagulation because the patient had symptoms of chronic ischemia. She exhibited satisfactory progress, without needing thrombolysis. Once the thrombus has been resolved, treatment should proceed with surgical release of thoracic outlet compression. Surgical treatment of cervical rib syndrome consists of resection, which can be accomplished via supraclavicular, posterior, transaxillary, or combined approaches, and via more recently described techniques such as video-assisted and transthoracic surgery with robotic assistance.^4^^-^6

Resection of the cervical rib and/or first rib via the supraclavicular approach provides access to the subclavian artery, which is of relevance in patients with aneurysms and/or thrombosis caused prolonged compression of the artery by the cervical rib, as was the case of the patient described in this report. The degree of integrity of the artery will determine whether repair or resection are needed.^3^ If the artery is only compressed, relieving the compression is a sufficient treatment.^3^ If there is greater arterial compromise or aneurysmal degeneration, a bypass is generally performed.^3^ In the case presented, release of adhesions from the right subclavian artery proved effective.

The transaxillary surgical approach is a safe technique involving reduced manipulation of the brachial plexus, which can achieve a lower incidence of perioperative complications related to nerve damage.^4^^,^^7^ It enables safe resection of cervical ribs and/or first ribs and is the approach most often used when concomitant resections are performed.^4^ It also produces better esthetic results than the supraclavicular approach.^4^ The disadvantage of this technique is that it does not offer adequate access to the subclavian artery. There is also a higher incidence of pneumothorax, probably due to the proximity of the pleura to the area dissected in the transaxillary approach.^4^ The combined approach should be used in cases in which a transaxillary approach does not provide an adequate view for resection of the cervical rib.^4^

Video-assisted surgery for resection of cervical ribs and/or first ribs offers better surgical access and enables the surgical team to clearly identify anatomic structures. It also allows for safer dissection and reduces the number of complications.^6^ One disadvantage of the video-assisted approach is difficulty in accessing the superior portions of the scalene muscles; only the inferior 2 cm can be resected.^6^ Video-assisted surgery is more expensive than conventional surgery, but is less expensive than robotically-assisted surgery.

Transthoracic robotically-assisted resection of the cervical rib is a minimally invasive technique that offers adequate visualization of the neurovascular and musculoskeletal structures. The improved view improves safety and enables complete surgical decompression. It also yields better esthetic results, since just three small surgical incisions are made, with the largest, at 15 mm, at the level of the armpit.^5^ However, this is a new technique with few cases reported and higher costs.

Use of anticoagulants for initial treatment of thrombosis is well-defined in treatment of TOS. Use of anticoagulants after resection of the first rib is controversial. Fairman et al.^8^ recommend preoperative thrombolysis, with the advantage of potential elimination of the risk of postoperative anticoagulation to treat Paget-Schroetter syndrome, which is the venous vascular form of TOS. Gelabert et al.^9^ describe use of anticoagulation with warfarin during the postoperative period and recommend against use of heparin because of the higher risk of bleeding. In the case reported, the patient was treated with warfarin preoperatively, with good response in terms of reduction of symptoms and no need for thrombolysis. Anticoagulation was not used during the postoperative period. The most recent studies demonstrate that patients are often discharged on aspirin alone during the postoperative period, with no need for anticoagulation.^8^

Immediate treatment of cervical rib syndrome is important to prevent long-term complications of neural and/or vascular compression.^10^ Asymptomatic patients in whom a cervical rib is found as an incidental diagnosis should be given guidance on the symptoms of neurovascular compression, so that they can seek appropriate treatment rapidly in the event that symptoms emerge.^2^

## CONCLUSIONS

Cervical rib syndrome is rare, but has great potential to become severe, causing significant morbidity if not treated adequately. We should remember mechanical compression as a possible cause of cases of arterial occlusion in young patients.

Resection of the cervical rib via a supraclavicular approach is a safe treatment that offers satisfactory access to the subclavian artery, good clinical results, and a favorable impact on the recovery of patients with cervical rib syndrome. In these cases, anticoagulants are indicated for initial treatment of thrombosis and anticoagulation is generally unnecessary during the postoperative period.

## References

[1] Ciorlin E, Araújo JD, Araújo JD, Brito CJ, Silva RM, Araújo EL (2020). Síndromes compressivas neurovasculares cervicotoracoaxilares (Síndrome do desfiladeiro).. Cirurgia vascular: cirurgia endovascular, angiologia..

[2] Henry BM, Vikse J, Sanna B (2018). Cervical rib prevalence and its association with thoracic outlet syndrome: a meta-analysis of 141 studies with surgical considerations. World Neurosurg.

[3] Daniels B, Michaud L, Sease F, Cassas KJ, Gray BH (2014). Arterial thoracic outlet syndrome. Curr Sports Med Rep.

[4] Jayaraj A, Duncan AA, Kalra M, Bower TC, Gloviczki P (2018). Outcomes of transaxillary approach to cervical and first-rib resection for neurogenic thoracic outlet syndrome. Ann Vasc Surg.

[5] Wybaillie E, Maene L, Cooreman F, Beelen R (2018). Robotically assisted transthoracic cervical rib resection. Ann Thorac Surg.

[6] Chan YC, Gelabert HA (2013). Hight-definition video-assisted transaxillary first rib resection for thoracic outlet syndrome. J Vasc Surg.

[7] Gelabert HA, Rigberg DA, O’Connell JB, Jabori S, Jimenez JC, Farley S (2018). Transaxillary decompression of thoracic outlet syndrome patients presenting with cervical ribs. J Vasc Surg.

[8] Fairman AS, Fairman RM, Foley PJ, Etkin Y, Jackson OA, Jackson BM (2020). Is routine postoperative anticoagulation necessary in all patients after first rib resection for paget-schroetter syndrome?. Ann Vasc Surg.

[9] Gelabert HA, Jimenez JC, Davis GR, Derubertis BG, O’Connell JB, Rigberg DA (2011). Early postoperative hemorrhage after first rib resection for vascular thoracic outlet syndrome. Ann Vasc Surg.

[10] Morel J, Pirvu A, Elie A, Gallet N, Magne JL, Spear R (2019). Functional results of cervical rib resection for thoracic outlet syndrome: impact on professional activity. Ann Vasc Surg.

