# Supplementary material for: Treatment of upper limb arterial occlusion caused by a cervical rib
Source: J Vasc Bras. 2021 Jun 16;20:e20200193. doi: 10.1590/1677-5449.200193 (PMC8218826; doi:10.1590/1677-5449.200193)
Supplement: Translation

# Translation: Tratamento de oclusão arterial em membro superior causada por costela cervical

**Authors:** Vanessa Aline Miranda Vieira Milagres, Roberto Lucas de Sena Avellar, Ana Paula Pires Silva, Pedro José Pires, Daniel Mendes Pinto

**Affiliations:** 1 Hospital Felício Rocho, Belo Horizonte, MG, Brasil.

**Keywords:** costela cervical, síndrome do desfiladeiro torácico, artéria subclávia, veia subclávia, plexo braquial

## Abstract

A síndrome da costela cervical ocorre quando o triângulo intercostoescalênico é ocupado por uma costela cervical, deslocando o plexo braquial e a artéria subclávia anteriormente, o que pode gerar dor e espasmo muscular. O objetivo deste estudo é discutir sobre o diagnóstico da síndrome da costela cervical e as possibilidades de tratamento. Este desafio terapêutico descreve a condução clínica e cirúrgica de uma paciente de 37 anos com obstrução arterial em membro superior causada por costela cervical.

## INTRODUÇÃO

A costela cervical é uma variação anatômica presente em 1% da população, a qual origina-se na apófise transversa da sétima vértebra cervical^1^. A maioria dos pacientes é assintomática, e somente cerca de 10% manifestam sintomas^1^. Os sintomas, quando presentes, são decorrentes da compressão de estruturas neurovasculares na região do desfiladeiro torácico, compreendendo a síndrome da costela cervical que representa um tipo de síndrome do desfiladeiro torácico (SDT). A costela cervical está presente em quase 30% dos casos da SDT^2^.

O tratamento cirúrgico da síndrome da costela cervical pode ser realizado por vias de acesso convencionais, como a abordagem supraclavicular, posterior, transaxilar ou combinada^3^^,^^4^. As técnicas de acesso mais recentemente descritas são a videoassistida e a transtorácica por robótica^5^^,^^6^.

O objetivo deste estudo é discutir o diagnóstico da síndrome da costela cervical e as possibilidades de tratamento. Apresentamos o caso de uma paciente de 37 anos com obstrução arterial em membro superior causada por costela cervical, bem como sua condução clínica e cirúrgica. O protocolo foi aprovado pelo Comitê de Ética de nossa instituição (CAAE 35649620.2.0000.5125, parecer de aprovação nº 4.303.586).

## PARTE I – SITUAÇÃO CLÍNICA

Paciente de 37 anos, do sexo feminino, previamente hígida, admitida no pronto atendimento em setembro de 2019 com dor, palidez e parestesia em membro superior direito. Relatava início dos sintomas há 4 meses, com piora nas últimas 2 semanas. Ao exame físico do membro superior direito, apresentava palidez distal, pulso palpável em região supraclavicular direita, pulso braquial reduzido e ausência de pulsos radial e ulnar. Apresentava costela cervical palpável à direita. Foi realizado dúplex arterial do membro superior direito, que evidenciou trombos de aspectos crônicos em artérias radial e ulnar com oclusão. A radiografia da coluna cervical e do tórax mostrava presença de costela cervical à direita articulada. Diante dessa situação, havia algumas possibilidades terapêuticas:

1- Anticoagulação sistêmica com heparina;
2- Trombólise dirigida por cateter;
3- Cirurgia para ressecção da costela cervical.

## PARTE II – O QUE FOI FEITO

A paciente foi internada, foi realizado controle da dor e feita anticoagulação com enoxaparina a 1 mg/kg 12/12 h. A angiotomografia da aorta torácica e do membro superior direito identificou arco costal acessório cervical (C7) à direita, articulando-se anteriormente com o primeiro arco costal ipsilateral. Na artéria subclávia pérvia, destaca-se, no entanto, acentuada compressão dela entre o arco costal acessório supracitado e a clavícula ipsilateral, notadamente durante a abdução do membro superior direito (Figura 1). Demais segmentos da artéria subclávia direita pérvios não tinham evidência de compressão. Estabelecido o diagnóstico de SDT, a paciente recebeu alta com varfarina, apresentava exame de razão normatizada internacional de 2,39 e ácido acetilsalicílico, com orientação de realizar acompanhamento ambulatorial e programar cirurgia para ressecção da costela cervical.

**Figura 1:** Angiotomografia evidenciando costela cervical à direita e compressão da artéria subclávia.

Após 1 mês de anticoagulação terapêutica, em outubro de 2019, a paciente foi internada para tratamento cirúrgico eletivo da SDT. Foi realizado acesso com incisão supraclavicular como ilustrado na Figura 2, com identificação da costela cervical, da artéria subclávia e do plexo braquial (Figura 3).

**Figura 2:** Incisão em “colar” 2 cm acima da clavícula.

**Figura 3:** A seta amarela identifica a costela cervical. Reparo da artéria subclávia com *vessel loop* vermelho.

A paciente foi submetida à ressecção da costela cervical à direita (Figura 4), com dissecção e liberação de aderências da artéria subclávia direita e exploração do plexo braquial. O ato cirúrgico foi bem tolerado e sem intercorrências. A radiografia do tórax no perioperatório não apresentava pneumotórax. No pós-operatório, a paciente evoluiu bem, com melhora da dor, sensibilidade e motricidade preservadas em membros superiores, pulsos radiais cheios e simétricos. A paciente recebeu alta no primeiro dia de pós-operatório em uso de ácido acetilsalicílico 100 mg ao dia. No seguimento de 6 meses, a paciente encontrava-se assintomática e com boa perfusão no membro superior direito.

**Figura 4:** (A) Costela cervical; (B) Artéria subclávia após ressecção da costela cervical.

## DISCUSSÃO

Este trabalho chama atenção para a oclusão arterial em pacientes jovens que pode ser causada por compressões mecânicas. Cerca de 50% dos pacientes com síndrome da costela cervical apresentam compressão arterial^2^. O tratamento por via cervical é adequado nesta situação, pois permite o acesso seguro ao plexo braquial, à artéria subclávia e à costela cervical.

A SDT descreve os sintomas causados pela compressão de estruturas neurovasculares na região do desfiladeiro torácico^5^. A síndrome da costela cervical ocorre quando o triângulo intercostoescalênico é ocupado por uma costela cervical, deslocando o plexo braquial e a artéria subclávia anteriormente, gerando dor e espasmo muscular^1^.

O tratamento da maioria dos pacientes com SDT é clínico, realizado com analgésicos, anti-inflamatórios, benzodiazepínicos e mudanças posturais^1^. No caso apresentado, o tratamento clínico foi feito inicialmente com analgésicos e anticoagulação terapêutica porque a paciente apresentava oclusão arterial. A cirurgia tem indicação formal em 15% dos casos, e a maioria das operações para SDT ocorre em paciente com compressão neurogênica^1^. A presença de costela cervical, alterações ósseas sintomáticas e complicações vasculares, como aneurismas e tromboses, tem indicação cirúrgica mandatória^1^^,^^2^. Quando o trombo está presente, a trombólise dirigida por cateter é o tratamento inicial preferido por Daniels et al.^3^. No caso em questão, optamos por tratar inicialmente com anticoagulação, pelo fato de a paciente encontrar-se com sintomas crônicos de isquemia. A paciente apresentou evolução satisfatória sem necessidade de trombólise. Uma vez que o trombo é removido, deve-se seguir a descompressão cirúrgica do desfiladeiro torácico. O tratamento cirúrgico da síndrome da costela cervical envolve a sua ressecção, que pode ser realizada através de abordagem supraclavicular, posterior, transaxilar ou combinada, e as vias mais recentemente descritas são a videoassistida e a transtorácica por robótica^4^^-^6.

A ressecção da costela cervical e/ou primeira costela por abordagem supraclavicular permite acesso à artéria subclávia, relevante nos pacientes com aneurisma e trombose causados por compressão prolongada da artéria pela costela cervical, como no caso da paciente descrita neste relato. A integridade da artéria determina se o seu reparo ou ressecção são necessários^3^. Se a artéria estiver apenas comprimida, a remoção da compressão é suficiente para o tratamento^3^. Quando há comprometimento maior da artéria ou degeneração aneurismática, o *bypass* geralmente é realizado^3^. No caso apresentado, a liberação de aderências da artéria subclávia direita foi eficaz para o tratamento.

A abordagem cirúrgica transaxilar é uma técnica segura, que envolve menor manipulação do plexo braquial, podendo resultar em menor incidência de complicações perioperatórias associadas a lesões nervosas^4^^,^^7^. Também permite a ressecção de costelas cervicais e/ou primeiras costelas com segurança, sendo o acesso mais utilizado para resseções concomitantes^4^. Além disso, possui melhor resultado estético quando comparada à abordagem supraclavicular^4^. Como desvantagem, essa técnica não permite acesso adequado à artéria subclávia. Há também maior incidência de pneumotórax, provavelmente devido à proximidade da pleura com a área de dissecção na abordagem transaxilar^4^. A abordagem combinada deve ser usada em casos de visualização inadequada por via transaxilar para ressecção de costela cervical^4^.

A cirurgia videoassistida para ressecção de costelas cervicais e/ou primeiras costelas fornece melhor acesso cirúrgico e permite a identificação clara de estruturas anatômicas para a equipe cirúrgica. Além disso, proporciona uma dissecção mais segura e reduz o número de complicações^6^. Como desvantagem, pela abordagem videoassistida é difícil acessar as porções superiores dos músculos escalenos; apenas os 2 cm inferiores podem ser ressecados^6^. A cirurgia videoassistida tem maior custo que a cirurgia convencional, porém é mais barata quando comparada à cirurgia robótica.

A ressecção da costela cervical transtorácica assistida por robô é uma técnica minimamente invasiva que proporciona uma visualização adequada das estruturas neurovasculares e musculoesqueléticas. Ao melhorar a visualização, promove maior segurança e completa descompressão cirúrgica. Além disso, possui um melhor resultado estético, pois são realizadas apenas três pequenas incisões cirúrgicas, sendo a maior de 15 mm ao nível da axila^5^. Entretanto, é uma técnica nova com poucos casos relatados e maior custo.

O uso de anticoagulantes para tratamento inicial da trombose é bem definido no tratamento da SDT. O uso de anticoagulantes após a ressecção da primeira costela é controverso. Fairman et al.^8^ preconizam a trombólise no pré-operatório com a vantagem da eliminação potencial do risco da anticoagulação pós-operatória para tratamento da síndrome de Paget-Schroetter, que representa a classificação do tipo vascular venoso da SDT. Gelabert et al.^9^ descrevem o uso de anticoagulantes no pós-operatório com varfarina e desaconselham o uso de heparina devido a maior risco de sangramento. No caso relatado, a paciente recebeu tratamento com varfarina no pré-operatório, com boa resposta na redução dos sintomas, sem necessidade de trombólise. Não foi realizada anticoagulação no pós-operatório. Os estudos mais recentes demonstram que, no pós-operatório, os pacientes recebem alta frequente apenas com aspirina, sem necessidade de anticoagulação^8^.

O tratamento imediato da síndrome da costela cervical é importante para prevenir complicações a longo prazo decorrentes da compressão neural e/ou compressão vascular^10^. Os pacientes assintomáticos, com diagnóstico incidental de costela cervical, devem ser orientados sobre os sintomas decorrentes da compressão neurovascular para que, em caso de desenvolvimento de sintomas, esses pacientes tenham tratamento rápido e apropriado^2^.

## CONCLUSÃO

A síndrome da costela cervical é rara, mas com grande potencial de gravidade, resultando em morbidade significativa se não tratada adequadamente. Devemos lembrar da possibilidade de compressões mecânicas nos casos de oclusão arterial em pacientes jovens.

A ressecção da costela cervical por acesso supraclavicular é um tratamento seguro, com acesso satisfatório à artéria subclávia, bons resultados clínicos e impacto favorável na recuperação do paciente com síndrome da costela cervical. Nesses casos, o uso de anticoagulantes para tratamento inicial da trombose é indicado, e geralmente não é necessária anticoagulação no pós-operatório.
